# IgA nephropathy management: what does the future hold?

**DOI:** 10.1016/j.kisu.2026.01.001

**Published:** 2026-07-20

**Authors:** Sydney C.W. Tang, Heather N. Reich

**Affiliations:** 1 Division of Nephrology, Department of Medicine, School of Clinical Medicine, The University of Hong Kong, Queen Mary Hospital, Hong Kong, China; 2 Division of Nephrology, University Health Network, Toronto, Ontario, Canada; 3 Temerty Faculty of Medicine, University of Toronto, Toronto, Ontario, Canada

**Keywords:** future treatments, guidelines, IgA1, IgA nephropathy

## Abstract

Increased understanding of the pathogenesis of IgA nephropathy (IgAN) has led to the development of new investigational agents tailored to this disease. The identification of surrogate markers of early treatment benefit has facilitated regulatory approval of these novel therapies. The surge in available data from several completed trials has prompted the recent revision of the Kidney Disease: Improving Global Outcomes guidelines for IgAN. Two agents have been fully approved for use in patients with IgAN in some regions: Nefecon, an oral targeted-release formulation of budesonide, and sparsentan, a dual endothelin A and angiotensin II receptor antagonist. Iptacopan and atrasentan are conditionally approved on the basis of proteinuria data; full approval will be evaluated once data on kidney function are available. With the potential future expansion of the IgAN treatment armamentarium, a dual approach to IgAN management is highly likely, addressing the overproduction of galactose-deficient IgA1 and downstream consequences of deposition of nephritogenic immune complexes, as well as considering supportive strategies targeting common non–disease-specific ongoing nephron loss. Combination therapies with different classes of drugs will likely become the new standard of care for chronic kidney disease as they target distinct pathogenic mechanisms or “hits” (namely, hemodynamic, immune, galactose-deficient IgA1–generating, inflammatory, and fibrotic processes involved in IgAN and chronic kidney disease progression). To improve personalized care for patients with IgAN, further research is needed to identify and validate noninvasive biomarkers for diagnosis, prognosis, treatment selection, and monitoring response.

Patients with IgA nephropathy (IgAN) are at risk of progression to advanced chronic kidney disease (CKD) within their lifetime despite optimized supportive care, with a significant proportion progressing to kidney failure within 10 to 15 years (Sim JJ, Chen Q, Chang JM, et al. ESKD and CKD progression among a diverse immunoglobulin A nephropathy [IgAN] population [abstract]. Presented at: American Society of Nephrology Kidney Week. November 1–5, 2023; Philadelphia, PA. Abstract TH-PO615).^1^, ^2^, ^3^ Depending on estimated glomerular filtration rate (eGFR) and age at diagnosis, kidney failure can be delayed if a rate of eGFR loss <1 ml/min per 1.73 m^2^ per year (i.e., the physiological attrition rate) is maintained.^1^ Given the negative impact that IgAN can have on a patient’s quality of life and the burden that progression to kidney failure and potential lifelong dialysis or transplantation can cause, early diagnosis and better management of IgAN, including novel treatment options, is required (Tyagi N, Aasaithambi S, Chauhan J, et al. Patient insights for immunoglobulin A nephropathy [IgAN] using social media listening [poster]. Presented at: International Society for Pharmacoeconomics and Outcomes Research – Europe. November 17–20, 2019; Copenhagen, Denmark. Poster PUK32; and George AT, Lafayette R, Tang S, et al. Treatment goals from the perspective of immunoglobulin A nephropathy patients - results from a real-world study [abstract]. Presented at: World Congress of Nephrology. April 13–16, 2024; Buenos Aires, Argentina. Abstract 1163).^4^, ^5^, ^6^, ^7^

**Key Learning Points**

• Significant strides have been made in improving our understanding of IgA nephropathy (IgAN) pathophysiology and surrogate markers of treatment benefit, which has led to novel drug discoveries and changes in guidance on how IgAN is managed, and opened up avenues of research into further novel therapies. This new evidence has prompted the revision of the Kidney Disease: Improving Global Outcomes guidelines for IgAN.
• There are still many unanswered questions as to how management of this chronic progressive disease can be optimized, which will require further clinical and real-world research. There is particular interest in the use of noninvasive biomarkers for screening patients, selecting appropriate treatments, and monitoring response to them to provide an individualized, patient-centered approach to management.

Over the past decade, the treatment landscape for IgAN has evolved rapidly because of our increasing understanding of the pathogenesis of IgAN.^8^ Owing to increased clinical research on IgAN, the rate of decline in eGFR and proteinuria reduction have been identified as surrogate end points that can be used to assess a treatment’s effect on progression to kidney failure.^8^, ^9^, ^10^ More information regarding these topics can be found in other articles within this supplement.

This review will broadly discuss clinical guidelines, the expected changes that we are likely to see in IgAN management in the coming years, and the remaining challenges and unmet needs in this therapeutic area.

## Clinical Guidelines

Key clinical guidelines for the management of IgAN are published by Kidney Disease: Improving Global Outcomes (KDIGO), an organization that develops and implements evidence-based global clinical practice guidelines with the aim of improving the care of patients and their outcomes.^11^ The KDIGO guidelines are published as open access documents and are freely accessible on the KDIGO website. International accessibility of recommended treatments is an important consideration and a point of discussion in the approaches to treatments outlined in the revised guidelines. Peer-reviewed published evidence is considered and reported on the basis of a structured and transparent evaluation system, including a month-long open consultation process.

Guidelines are intended to improve clinical and public health outcomes by encouraging best practice among health care professionals, aiding policy makers in improving the quality of care provision, and closing the gap between current clinical practice and the latest scientific evidence (Table 1).^12^, ^13^, ^14^, ^15^ Most guidelines rely heavily on results of trials that usually study relatively homogeneous populations, whereas in real-life settings, patients may have complex conditions and comorbidities that require polypharmacy.^13^^,^^14^ Therefore, guidelines based largely on evidence from clinical trial populations may be challenging to adapt to the individual needs of patients and their priorities.^13^^,^^14^ In addition, because of the rigorous process that is required to develop guidelines, they may be published years after evidence has become available, which may delay the widespread adoption of newer treatments.^12^^,^^14^^,^^16^

**Table 1:** Potential benefits and limitations of clinical guidelines^12^, ^13^, ^14^, ^15^

Category	Health care professionals	Patients	Health care systems
Potential benefits	Improve quality of clinical decisions and consistency of care. Evidence-based guidelines clarify which interventions are of proven benefit and document the quality of supporting data. Can greatly assist clinicians who are uncertain about how to proceed, challenge outdated practices, and provide authoritative recommendations that reassure practitioners of the appropriateness of their treatment strategies. Critical appraisal of the evidence can identify design flaws in existing studies and gaps in evidence, which can provoke change and refocus research. Support quality improvement activities.	Improve the consistency of care. Promote interventions of proven benefit and discourage ineffective ones, which may reduce morbidity and mortality and improve quality of life. Draw attention to underrecognized health problems, clinical services, and preventive interventions and to neglected and high-risk patient populations.	Effective in improving efficiency, often by standardizing care, and optimizing value for money. Publicizing adherence to guidelines may improve public image, highlighting commitment to excellence and quality while promoting goodwill, political support, and revenue in some health care systems.
Potential limitations	Clinicians often find them inconvenient and time-consuming to use. Efforts to summarize in figure format risks losing nuance and opportunities to tailor decisions to individual patients. Conflicting guidelines from different professional bodies can cause confusion and frustration. Outdated recommendations may perpetuate outmoded practices and technologies. Prescriptive guidelines may negatively impact clinicians’ independent decision-making and oversimplify treatment. Auditors and managers may judge the cost and quality of care that clinicians provide unfairly if based on criteria from outdated guidelines. Biases of authors and sponsors of guidelines can never be fully eliminated.	Blanket recommendations, rather than a menu of options or recommendations for shared decision-making, ignore patients' personal circumstances, medical history, and patients’ preferences, leading to reduced individualized care for patients. Patients' needs may not be the only priority. Suboptimal practices, from the patient's perspective, may be required to help control costs, serve societal needs, or protect interests if following them escalates utilization, compromises operating efficiency, or wastes limited resources.	Updating guidelines takes time; research may be even further advanced by the time they are published. Inapplicability to local settings and lack of active user involvement. Scientific evidence for recommendations can be lacking, with only a small subset being tested appropriately in well-designed studies. The value judgments made by guideline development groups may be the wrong choice for individual patients or varied health care systems. Recommendations are influenced by the opinions and clinical experience of the guideline development group.

### KDIGO guidelines

The first guidelines by KDIGO for glomerulonephritis were published in 2012; this summarized recommendations for 12 distinct diseases, including IgAN, and was the most extensive set of guidelines in KDIGO history at the time.^17^^,^^18^ However, the authors acknowledged that, based on the quality of evidence that was available at the time of publication, a high proportion of IgAN-related statements were suggestions rather than recommendations.^17^

#### KDIGO 2021 clinical practice guidelines for the management of glomerular diseases

**Figure 1:**
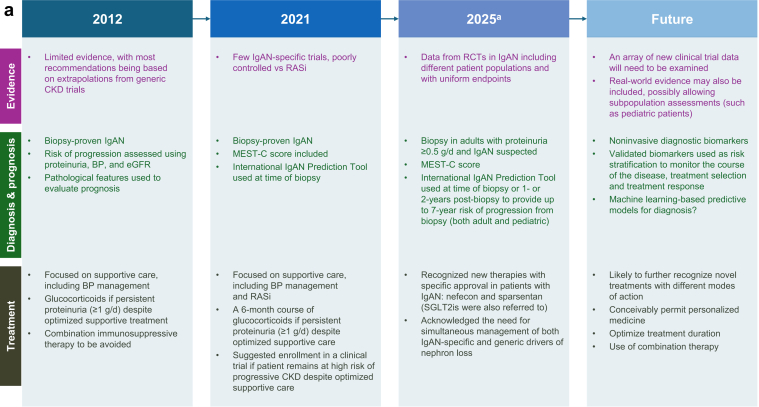

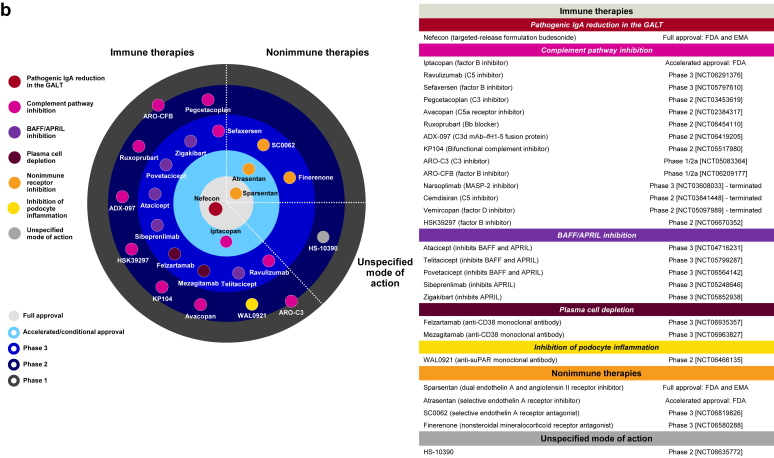
**(a) Kidney Disease: Improving Global Outcomes (KDIGO) guideline summary and what future KDIGO guidelines may encompass.** (**b**) Approved therapies^28^, ^29^, ^30^, ^31^, ^32^, ^33^ and multiple innovative therapeutic options undergoing clinical trials that are providing new insights in the management of IgA nephropathy (IgAN). ^a^This update takes into consideration new evidence from randomized controlled trials published from 2021 through April 2023. APRIL, a proliferation-inducing ligand; ARO-CFB, xxx; BAFF, B-cell–activating factor; BP, blood pressure; CD, cluster of differentiation; CKD, chronic kidney disease; eGFR, estimated glomerular filtration rate; EMA, European Medicines Agency; FDA, US Food and Drug Administration; GALT, gut-associated lymphoid tissue; mAb, monoclonal antibody; MASP-2, mannan-binding lectin-associated serine protease-2; MEST-C, mesangial hypercellularity, endocapillary hypercellularity, segmental glomerulosclerosis, tubular atrophy/interstitial fibrosis, and crescents; RASi, renin–angiotensin system inhibitor; RCT, randomized controlled trial; SGLT2i, sodium-glucose cotransporter-2 inhibitor; suPAR, soluble urokinase plasminogen activator receptor.

Because of a lack of data on IgAN-specific therapies at the time, the focus of the “KDIGO 2021 Clinical Practice Guideline for the Management of Glomerular Diseases” was on optimized supportive care (Figure 1a).^19^ This included the management of blood pressure in all patients with glomerular disease, based on extensive evidence in the general population with CKD.^19^ In patients with proteinuria >0.5 g/d, the guidelines recommended the use of either an angiotensin-converting enzyme inhibitor or an angiotensin II receptor blocker, irrespective of whether they have hypertension.^19^ It was also recommended that patients who remained at high risk of progressive CKD, despite maximal support, be considered for a 6-month course of glucocorticoid therapy.^19^ However, this was a “weak” recommendation because of the significant risk of toxicity noted with this therapy.^19^ This was largely based on data from the Therapeutic Evaluation of Steroids in IgA Nephropathy Global (TESTING) trial.^20^ At the time of the publication of the guidelines, the only data available were from the TESTING high-dose glucocorticoid protocol.^20^ The study had been paused and the protocol redesigned after an excess risk of serious adverse events—primarily from potentially preventable serious infections—was noted in patients receiving glucocorticoids. Nonetheless, the initial analysis was suggestive of efficacy with respect to proteinuria reduction and kidney outcomes. Because of the excess of adverse events and deaths observed, use of prednisone was only “suggested” (weak recommendation) despite moderate-level evidence (grade 2). Additional treatments, such as mycophenolate and hydroxychloroquine, were suggested in distinct patient populations, but no treatment specifically approved for the management of IgAN was available at this time.^19^ The 2021 guidelines also suggested that patients be offered the opportunity to enroll in a clinical trial if optimized supportive care alone had not helped reduce their risk of progression.^19^ Therefore, the emphasis was on prioritizing treatments with the lowest potential for toxicity and only managing the generic physiological response to IgAN-induced nephron loss.

#### Further updates to KDIGO clinical practice guidelines in IgAN

In 2025, an updated set of KDIGO guidelines was published.^11^ In the years since the 2021 guidelines were published, a substantial amount of data on therapies in patients with IgAN have become available (Table 2^20^, ^21^, ^22^, ^23^, ^24^, ^25^, ^26^, ^27^, ^28^, ^29^, ^30^, ^31^, ^32^, ^33^ and Figure 1b^28^, ^29^, ^34^, ^35^, ^36^, ^37^, ^38^, ^39^), with novel agents being granted approval in the population with IgAN in select geographic regions: Nefecon (an oral targeted-release formulation of budesonide), sparsentan (a dual endothelin A and angiotensin II receptor antagonist), and conditional approval of iptacopan (a complement factor B inhibitor) and atrasentan (a selective endothelin type A receptor antagonist).^3^^,^^28^, ^29^, ^30^, ^31^, ^32^, ^33^ These approvals were all based on published peer-reviewed data from rigorously designed, global, multicenter, phase 3 randomized clinical trials in patients with IgAN and included broadly similar study end points.^3^^,^^21^^,^^23^^,^^25^^,^^27^ The updated KDIGO 2025 guidelines also suggest that enrollment in clinical trials should continue to be considered for all patients with IgAN (i.e., not just those at risk of progression despite optimized support of care alone, as in the 2021 guidelines). This should enable research into new therapies to advance rapidly.^11^

**Table 2:** Overview of phase 3 trials of therapies with specific approval in patients with IgAN (Nefecon, sparsentan, iptacopan, and atrasentan) and/or with recommendations for their restricted use included in the updated KDIGO 2025 guidelines (dapagliflozin, empagliflozin, and systemic glucocorticoid)^20^, ^21^, ^22^, ^23^, ^24^, ^25^, ^26^, ^27^, ^28^, ^29^, ^30^, ^31^, ^32^, ^33^

Variable	NefIgArd^21^^,^^22^ (NCT03643965)	PROTECT^23^^,^^24^ (NCT03762850)	APPLAUSE-IgAN^25^^,^^26^ (NCT04578834)	ALIGN^27^^,^^28^ (NCT04573478)	DAPA-CKD^29^^,^^30^ (NCT03036150)	EMPA-KIDNEY^31^^,^^32^ (NCT03594110)	STOP-IgAN^33^	TESTING^20^
Agent	Nefecon	Sparsentan	Iptacopan^a^	Atrasentan^a^	Dapagliflozin	Empagliflozin	Systemic glucocorticoid	Systemic glucocorticoid
Mode of action	Targeted-release formulation budesonide	Dual endothelin A and angiotensin II receptor antagonist	Factor B inhibitor	Selective endothelin A inhibitor	SGLT2i	SGLT2i	Systemic immunosuppression	Systemic immunosuppression
Trial design	Phase 3, randomized, placebo controlled	Phase 3, randomized, active controlled	Phase 3, randomized, placebo controlled	Phase 3, randomized, placebo controlled	Phase 3, randomized, placebo controlled	Phase 3, randomized, placebo controlled	Phase 3, randomized, open label	Phase 3, randomized, placebo controlled
Patients and dosing	Aged ≥18 y; biopsy-confirmed IgAN; eGFR 35–90 ml/min per 1.73 m^2^; persistent proteinuria. Nefecon, 16 mg/d^b^ (n = 182); placebo^b^ (n = 182)	Aged ≥18 y; biopsy-confirmed IgAN; proteinuria ≥1 g/d; eGFR ≥30 ml/min per 1.73 m^2^; BP ≤150/100 mm Hg. Sparsentan, 400 mg once daily (n = 202); irbesartan, 300 mg once daily (n=202)	Aged ≥18 y; biopsy-confirmed IgAN; UPCR ≥1 g/g, eGFR ≥30 ml/min per 1.73 m^2^. Iptacopan, 200 mg twice daily^b^ (n = 125); placebo^b^ (n = 125)	Aged ≥18 y; biopsy-confirmed IgAN; total urinary protein excretion ≥1 g/d, eGFR ≥30 ml/min per 1.73 m^2^. Atrasentan, 0.75 mg once daily (n = 135); placebo (n = 135)	Aged ≥18 y; general CKD (with prespecified analysis in patients with IgAN); eGFR 25–75 ml/min per 1.73 m^2^; UACR 200–5000 mg/g. Dapagliflozin, 10 mg once daily^b^ (n = 137); placebo^b^ (n = 133)	Aged ≥18 y; general CKD (including 817 patients with IgAN); eGFR ≥20–<45 or ≥45–<90 ml/min per 1.73 m^2^; UACR 200 mg/g. Empagliflozin, 10 mg once daily^b^; placebo^b^	Aged ≥18 y; biopsy-confirmed IgAN; protein excretion >0.75 g/d. Supportive care only (n = 80); supportive care plus immunosuppression (n = 82)^c^	Aged ≥18 y; biopsy-confirmed IgAN; eGFR 20–120 mL/min per 1.73 m^2^; protein excretion >1 g/d. Oral methylprednisolone (0.6–0.8 mg/kg per day^d^ (n = 136); placebo (n = 126)
Mean absolute difference in eGFR, ml/min per 1.73 m^2^	At 9 mo: 5.21 (95% CI, 3.35–7.58) At 24 mo: 5.89 (95% CI, 3.35–9.15)	At 110 wk: 3.7 (95% CI, 1.5–6.0) At 114 wk: 2.9 (95% CI, 0.5–5.3)	NA	NA	No difference	NA	No difference	5.15 (95% CI, 0.42–9.89; *P* = 0.03)
Difference in eGFR slope, ml/min per 1.73 m^2^ per yr	Total slope at 24 mo: 2.95 (95% CI, 1.67–4.58; *P* < 0.0001)	Chronic slope: 1.1 (95% CI, 0.1–2.1; *P* = 0.037) Total slope: 1.0 (95% CI, –0.03 to 1.94; *P* = 0.058)	NA	NA	NA	Chronic slope: 1.78 (95% CI, 1.13–2.44) Total slope: 1.14 (95% CI, 0.54–1.75)	NA	NA
Proteinuria data	At 9 mo: 30.0% (95% CI, 19.9%–38.8%)^e^ At 24 mo: 30.1% (95% CI, 16.4%–41.5%)^e^	At wk 110: 40% (geometric least-squares mean ratio: 0.60 [95% CI, 0.50–0.72])^e^	At 9 mo: 38.3% (95% CI, 26.0%–48.6%; *P* < 0.001)^e^	At wk 36: geometric mean between-group reduction 36.1% (95% CI, −44.6% to −26.4%; *P* < 0.001)	NA	NA	NA	Mean time-averaged proteinuria absolute difference: –0.99 g/d (95% CI, –1.34 to −0.64; *P* < 0.001)
Hematuria data	Odds ratio: 2.5 (95% CI, 1.6–4.1; *P* = 0.0001)	NA	At 9 mo, hematuria no longer present with iptacopan vs. placebo group: 38.7% vs. 16.3%	NA	NA	NA	Disappearance of microhematuria, n (%) Supportive care only: 9 (16); plus immunosuppression: 24 (42); odds ratio: 3.73 (95% CI, 1.52–9.14; *P* = 0.004)	Disappearance of hematuria, absolute risk difference: 23.2% (95% CI, 5.8%–38.7%; *P* = 0.01)
Safety data	Nefecon vs. placebo: Peripheral edema: 31 (17%) vs. 7 (4%); hypertension: 22 (12%) vs. 6 (3%); muscle spasms: 22 (12%) vs. 7 (4%); acne: 20 (11%) vs. 2 (1%); and headache 19 (10%) vs. 14 (8%)	Sparsentan vs. irbesartan: COVID-19: 53 (26%) vs. 46 (23%); hyperkalemia: 32 (16%) vs. 26 (13%); peripheral edema: 31 (15%) vs. 24 (12%); dizziness: 30 (15%) vs. 13 (6%); headache: 27 (13%) vs. 26 (13%); hypotension: 26 (13%) vs. 8 (4%); and hypertension: 22 (11%) vs. 28 (14%)	Iptacopan vs. placebo: COVID-19: 31 (14%) vs. 37 (17%); upper respiratory tract infection: 20 (9%) vs. 16 (7%); nasopharyngitis: 11 (5%) vs. 16 (7%); headache: 9 (4%) vs. 12 (5%); and hypertension: 4 (2%) vs. 13 (6%)	Atrasentan vs. placebo: Any severe AEs: 12 (7%) vs. 10 (6%); any AEs leading to discontinuation of atrasentan or placebo: 6 (4%) vs. 6 (4%); AEs of special interest: anemia^f^ 14 (8%) vs. 4 (2%); fluid retention 19 (11%) vs. 14 (8%); vasodilatation or hypotension 10 (6%) vs. 7 (4%)	Dapagliflozin vs. placebo: AEs leading to discontinuation of study drug: 6 (4%) vs. 7 (5%); serious AEs, including death: 22 (16%) vs. 34 (26%)	Empagliflozin vs. placebo^g^: Serious hyperkalemia: 15 (2%) vs. 20 (3%); serious AKI: 12 (1%) vs. 20 (3%); bone fracture: 24 (3%) vs. 21 (3%); serious UTI: 7 (1%) vs. 8 (1%); symptomatic dehydration: 12 (1%) vs. 13 (2%)	Supportive care only vs. supportive care plus immuno suppression: ≥1 SAE: 21 vs. 29; total No. of SAEs: 29 vs. 33; total No. of events of infection: 111 vs. 174; total No. of SAEs of infection: 3 vs. 8	Methylprednisolone vs. placebo: total SAE, n (events): 20 (28) vs. 4 (4); SAE of infection: 11 (13) vs. 0 (0)
References	Lafayette *et al.*^21^	Rovin *et al.*^22^	Perkovic *et al.*^23^	Heerspink *et al.*^24^	Wheeler *et al.*^25^	EMPA-KIDNEY Collaborative Group.^26^	Rauen *et al.*^27^	Lv J *et al.*^20^

AE, adverse event; AKI, acute kidney injury; BP, blood pressure; CI, confidence interval; CKD, chronic kidney disease; COVID-19, coronavirus disease 2019; eGFR, estimated glomerular filtration rate; IgAN, IgA nephropathy; KDIGO, Kidney Disease: Improving Global Outcomes; NA, not available; NefIgArd, Nefecon in Patients with Primary IgA Nephropathy at Risk of Progressing to End-Stage Renal Disease; SAE, serious adverse event; SGLT2i, sodium-glucose cotransporter-2 inhibitor; STOP-IgAN, Supportive Versus Immunosuppressive Therapy for the Treatment of Progressive IgA Nephropathy; TESTING, Therapeutic Evaluation of Steroids in IgA Nephropathy Global; UACR, urine albumin-creatinine ratio; UPCR, urine protein-creatinine ratio; UTI, urinary tract infection.

a Not included in the KDIGO 2025 guidelines as phase 3 data were not available at the time of evaluation.

b Plus optimized supportive care.

c Level of eGFR >60 ml/min per 1.73 m^2^ received a glucocorticoid with prednisolone and <60 ml/min per 1.73 m^2^ received prednisolone with cyclophosphamide followed by azathioprine.

d With a maximum dose of 48 mg/d.

e Percentage reduction in UPCR versus comparator.

f No patient with anemia received a blood transfusion.

g AEs >1% reported for patients with glomerular disease.

Only therapeutic agents where full data have been described and published in peer-reviewed journals are eligible for inclusion in the KDIGO guidelines. Treatments may be approved by regulatory agents in advance of publication, but evidence must be publicly available to be assessed and graded before inclusion in these guidelines. Accordingly, the results of 2 randomized, controlled, double-blind, large-scale trials of Nefecon and sparsentan with 2-year eGFR data were published and incorporated into the KDIGO 2025 guidelines.^11^

The availability of these newer agents has also led to the possibility of differentiating treatments based on their ability to address the immunopathogenic drivers of nephron loss versus the generic responses to IgAN-induced nephron loss, as described in the updated guideline.^11^ In terms of interventions that have published data showing they address the immunopathogenesis of IgAN, only Nefecon and systemic glucocorticoids were eligible for inclusion. The updated 2025 guidelines also include recent data published describing the efficacy and risk profile of reduced-dose glucocorticoids in the TESTING study with antimicrobial prophylaxis against *Pneumocystis jirovecii*.^40^ The KDIGO 2025 guidelines also highlight the evidence supporting Nefecon as a first choice over systemic glucocorticoids in regions where it is available.^11^ Additional real-world data will be required to help determine whether certain clinical circumstances mandate an approach that favors systemic glucocorticoids versus targeted Nefecon therapy or vice versa.

In managing the generic responses to IgAN-induced nephron loss, the updated KDIGO 2025 guidelines include data regarding the use of sodium-glucose cotransporter-2 inhibitors with an optimized maximally tolerated dose of renin-angiotensin system inhibitor or the dual endothelin A and angiotensin II receptor antagonist sparsentan.^11^ The overarching goal of these treatments is to help optimize blood pressure control, reduce glomerular hyperfiltration, and limit the impact of proteinuria on the interstitium, as well as cardiovascular risk reduction. The suggestion to use a sodium-glucose cotransporter-2 inhibitor is based on data generated from studies of dapagliflozin and empagliflozin in patients with established CKD (Table 2) and data regarding the simultaneous use of sodium-glucose cotransporter-2 inhibitor and sparsentan with an optimized renin–angiotensin system inhibitor are not well established.^29^^,^^31^

Given the availability of disease-specific treatments, the updated KDIGO 2025 guidelines highlight the importance of simultaneously addressing both of these drivers of nephron loss when managing patients with IgAN.^11^ Previously, once patients achieved proteinuria <1 g/d with supportive care, there was less impetus to add immunosuppressive agents given the less favorable risk:benefit profile of drugs such as glucocorticoids. With the recent treatment approvals, and with many new agents being investigated across a range of biologic targets (Figure 1b), along with UK National Registry of Rare Kidney Diseases data demonstrating unfavorable prognosis even for patients with proteinuria <1 g/d, parallel combination therapy to manage IgAN and prevent irreversible nephron loss is potentially becoming a feasible treatment strategy.

Guidelines are also important to encourage payers (including public agencies) to ensure the availability of novel agents with better efficacy and risk profiles. However, the reality is that if a novel targeted therapy is not available or inaccessible in countries with a particularly high burden of IgAN, more traditional approaches, such as treatment with systemic glucocorticoids, will remain important mainstays of treatment despite their risk profile.

## Emerging Treatment Landscape

Clinical trials investigating new therapeutic strategies for the treatment of IgAN are focusing on both immune and nonimmune therapies targeting a wide range of different pathways (Figure 1b).^3^^,^^8^^,^^41^ Among immune therapies being investigated are selective or combined inhibitors of a proliferation-inducing ligand (APRIL) and B-cell–activating factor (BAFF) (e.g., atacicept [NCT04716231],^42^ sibeprenlimab [NCT04287985],^43^ and telitacicept [NCT04291781]^44^).^45^, ^46^, ^47^ The relative benefit and safety of using APRIL inhibition versus combined inhibition of APRIL and BAFF signaling will only be determined by randomized trials and subsequent real-world experience. The anti–cluster of differentiation 38 B-cell–depleting agents felzartamab (NCT05065970^48^) and mezagitamab (NCT05174221^49^) are being explored after anti–cluster of differentiation 20 treatment (rituximab) failed to improve outcomes in patients with IgAN.^50^ Use of these agents will address the hypothesis that pathogenic galactose-deficient IgA1 (Gd-IgA1) and anti–Gd-IgA1 autoantibodies are actually produced by plasma cells or plasmablasts.^51^, ^52^, ^53^

Among the complement inhibitor class of immune therapies being assessed in patients with IgAN are those targeting the alternative pathway (e.g., iptacopan [NCT04578834],^26^ sefaxersen [NCT05797610],^54^ and pegcetacoplan [NCT03453619]^55^) and the terminal pathway (e.g., ravulizumab [NCT04564339]^56^).^57^ Early results are encouraging, but full information from randomized trials will be essential to evaluate efficacy and to distinguish the impact of inhibiting complement activation at different levels. Unfortunately, a biologically plausible treatment strategy—such as targeting the lectin pathway of complement activation with the mannan-binding lectin-associated serine protease-2 inhibitor narsoplimab (NCT02682407^58^)—does not always translate into meaningful changes when evaluated in a clinical trial setting.^59^ On the other hand, nonimmune therapies, such as endothelin receptor antagonists (e.g., atrasentan [NCT04573478],^28^ with accelerated US Food and Drug Administration approval for proteinuria reduction granted in April 2025, and SC0062 [NCT05687890]^60^), may help manage the consequences of IgAN-induced kidney damage rather than the source of the disease.^27^^,^^61^^,^^62^

These agents are currently being assessed in phase 2 or 3 clinical trials in IgAN and have reported reductions in proteinuria, whereas some have also reported slowing of kidney function loss. Several of the immune-mediated therapies have shown a reduction in levels of Gd-IgA1, but many were accompanied by overall reductions in total Ig, such as IgG, IgA, and IgM (Barratt J, Workeneh B, Kim SG, et al. A phase 1/2 trial of zigakibart in IgA nephropathy [poster]. Presented at: American Society of Nephrology Kidney Week. October 23–27, 2024; San Diego, CA. Poster FR-PO856).^45^^,^^46^ More clinical data are awaited for these emerging therapies, and not all are likely to become fully approved agents for the treatment of IgAN. Indeed, the failure of highly targeted therapies to reach approval may relate to limitations in our ability to identify which disease pathways are most relevant in individuals who may not be readily identified by using serum creatinine and proteinuria for study eligibility. For example, lectin pathway–mediated complement activation may only be an actionable and effective approach in a subgroup of patients in whom there are biomarkers available that reflect lectin activity. Expanding the therapeutic armamentarium and coupling this work to enhanced clinical and molecular biomarker evaluation may enable a tailored approach to managing individual patients. Overall, the burgeoning list of novel approaches implies a promising future in the treatment of IgAN.^63^

## Patient-Centered Care

As mentioned previously, applying learnings from trial data to the real-world clinical picture is always challenging because of the strict patient inclusion and exclusion criteria implemented in clinical trials.^13^^,^^14^ In clinical practice, there is a greater need to assess the suitability of a given treatment for a patient, as well as the optimal timing for treatment relative to the course of the patient’s disease. The updated KDIGO 2025 guidelines highlight the importance of matching individual patients to trial populations in terms of clinical risk of progression (e.g., a patient with persistent proteinuria who is at risk of progressive loss of kidney function),^11^ although the impact of patient clinical and demographic characteristics on their suitability for a given treatment regimen remains to be explored. Although their strict inclusion criteria may homogenize populations, the global nature of completed and ongoing trials of treatments for IgAN and the resulting diversity of their patient populations may go some way to addressing this concern.

When tailoring treatments for IgAN, a patient’s genetic background and environment may need to be considered, as they can impact a patient’s susceptibility to IgAN and impact the patient’s risk of progression to kidney failure.^11^^,^^64^, ^65^, ^66^, ^67^, ^68^, ^69^ For example, a genome-wide association study reported more risk loci in patients with East Asian heritage compared with patients with European heritage, which is matched by a higher incidence of IgAN reported in Asian versus European populations.^64^^,^^66^ In addition, patients from South Asia are reported to present with a more aggressive disease phenotype than that seen in European and East Asian populations, which is not thought to be associated with delayed diagnosis.^70^ Therefore, risk of disease progression needs to be appropriately recognized so as not to hinder appropriate disease management in vulnerable patient groups.

Furthermore, a patient’s ancestry may also influence their response to certain treatments. For example, tonsillectomy has mainly been found to improve kidney survival and result in partial or complete remission of hematuria and proteinuria in patients with IgAN in studies performed in Pacific Asian populations; however, no significant correlation was found between tonsillectomy and kidney function decline in European patients with IgAN.^71^, ^72^, ^73^, ^74^, ^75^, ^76^, ^77^ Currently, tonsillectomy is not widely practiced for IgAN outside of Japan, where it has been recommended in the Japanese Society of Nephrology guidelines with or without pulsed glucocorticoids; as such, the updated KDIGO 2025 guidelines do not recommend it as a treatment in non-Japanese patients.^11^ Similarly, evidence reporting the efficacy of mycophenolate in managing IgAN has been mixed, with randomized clinical trials having been conducted in both East Asian and non-Asian populations, and with benefits noted only in East Asian populations.^11^^,^^78^, ^79^, ^80^, ^81^, ^82^, ^83^, ^84^, ^85^ Certain interventions have also been reported only in single patient populations, such as hydroxychloroquine, which was studied in Chinese patients and found to reduce proteinuria by approximately 48% at 6 months.^11^^,^^86^^,^^87^ Tonsillectomy, mycophenolate, and hydroxychloroquine are discussed in the article “Management of IgA nephropathy and the expanding role of immunomodulation” by Jain and Rizk.^88^ It is important not to “dismiss” these studies but instead to recognize and try to understand the unique nature and clinical characteristics of the populations included. Furthermore, treatments that have been evaluated in single-patient populations should perhaps not be dismissed or avoided in other patient populations. Considering IgAN is a chronic disease, further work to evaluate all available treatment options across diverse populations over time is needed so that patient care can be optimized.

## Unmet Need for Biomarkers in IgAN

There are several clinical markers of disease progression in IgAN, including hypertension, decreased eGFR, sustained proteinuria, and histopathologic lesions.^9^ Histopathologic scoring of lesions using the Oxford MEST-C (mesangial hypercellularity, endocapillary hypercellularity, segmental glomerulosclerosis, tubular atrophy/interstitial fibrosis, and crescents) classification system is a good prognostic marker for IgAN.^89^^,^^90^ The International IgAN Prediction Tool combines MEST-C scoring with demographic and clinical parameters. This score provides the individualized risk of a 50% reduction in eGFR or end-stage kidney disease up to 7 years from diagnosis and can be used at diagnosis or at a landmark time 1 or 2 years after diagnostic biopsy.^91^^,^^92^

On the basis of these findings and recent evidence suggesting a link between Oxford MEST-C scores and treatment effects, it seems likely that the pathologic findings reflect biologic processes that may help differentiate treatments for individual patients and ultimately help personalize management strategies for IgAN.^93^, ^94^, ^95^ For example, a biopsy that displays high levels of complement deposition would suggest that the patient may be more likely to respond to an agent that targets the complement system. Unfortunately, patients included in clinical trials tend to have had their biopsies performed a few years before the start of the trial, making it challenging to use this information to guide treatment selection.^21^^,^^25^^,^^96^ Therefore, noninvasive biomarkers are required to help select the right treatment for the right patient. More information regarding these topics can be found in articles within this supplement entitled “Management of IgA nephropathy and the expanding role of immunomodulation” by Canetta and Reich^97^ and “The expanding role of biomarkers in the management of IgA nephropathy” by Jain and Rizk.^88^

## Unanswered Questions and Unmet Needs in IgAN

### Optimal treatment regimen for patients with IgAN

Although several positive advances have been made, there are unanswered questions that remain. One question relates to the optimal duration of therapy to derive the most benefit from treatment interventions. As long-term follow-up data have become available, a recurrence of proteinuria has been observed once the initial treatment regimen has been completed.^20^^,^^21^^,^^98^ The TESTING trial assessed the use of oral methylprednisolone in patients with IgAN over a total treatment period of 6 to 8 months, and although an initial reduction in proteinuria was observed, this was no longer evident 36 months after randomization.^20^^,^^98^ Observational data indicate that the longer the duration of proteinuria remission, the greater the benefit on kidney survival.^99^ In the TESTING trial, kidney survival benefit remained evident despite recurrence of proteinuria, but it may have been even greater if a more sustainable treatment option was used that could have been continued at effective doses beyond 1 year. In a long-term study of mycophenolate in Chinese patients, a similar kidney survival benefit was observed despite recurrence of proteinuria after treatment cessation.^79^ Because of the chronic nature of IgAN, and the fact that current treatments are not cures, this effect is not unexpected and raises the possibility of whether sequential therapies, extended courses of treatment, or use of a lower maintenance dose is required to manage patients with IgAN. Recent data from the Nefecon in Patients with Primary IgA Nephropathy at Risk of Progressing to End-Stage Renal Disease (NefIgArd)^22^ open-label extension study have shown that similar treatment benefit with Nefecon was observed in both eGFR and proteinuria after 9 months of treatment, irrespective of whether the patients had previously received Nefecon treatment in the main phase 3 study, suggesting sequential treatment with Nefecon may be of benefit to the patient (Lafayette R, Kristensen J, Jones R, et al. NefIgArd open-label extension: efficacy and safety of nefecon in patients with IgAN who completed the 2-year phase 3 trial [abstract]. Presented at: American Society of Nephrology Kidney Week. October 23–27, 2024; San Diego, CA. Abstract FR-OR56). Extended Nefecon treatment, beyond a 9-month treatment course, is also being assessed in the NefXtend trial (NCT06712407).^100^ All current IgAN treatments require longer-term efficacy and safety data to fully understand what regimen would be best and in which patients.

### Clarifying the importance of the mechanism of proteinuria reduction with newer agents

Although proteinuria is associated with kidney outcomes and is considered a “reasonably likely” surrogate end point for progression to kidney failure in IgAN by the US Food and Drug Administration, changes in proteinuria can be attributed to a modification of immunopathogenic pathways of disease activity, changes in glomerular hemodynamics, or impact on glomerulosclerosis and interstitial fibrosis.^8^, ^9^, ^10^, ^11^^,^^101^^,^^102^ It is plausible that the most durable and clinically meaningful impact on the trajectory of disease in IgAN will be evident in trials of treatments that most directly impact immune-complex formation and glomerular inflammation. However, it is equally important to consider the impact of novel interventions on CKD progression in conditions outside of IgAN, particularly in patients with established irreversible kidney injury and moderate kidney function impairment. Further research is required to fully understand the importance of proteinuria remission duration with newer agents and how to best prioritize and combine supportive and immunologic strategies.

### Therapeutic management at different clinical stages of IgAN

Patients with IgAN who have experienced benefits with recently approved therapies still demonstrate a decline in eGFR that is likely to be greater than that observed in patients with general CKD or in healthy populations.^21^^,^^23^^,^^103^, ^104^, ^105^ This raises the question of what strategies can be implemented at the earliest stages to further delay or halt the progression of IgAN. For example, the optimal sequencing of disease-modifying strategies remains to be defined. A more potent therapy may be best suited for initiation earlier in the disease course if a high-risk patient can be appropriately identified. Some of the novel investigational therapies, such as those targeting APRIL/BAFF inhibition and complement inhibition, are associated with significant changes in immune pathways impacted by IgAN; however, further safety and real-world toxicity data are required before any attempt to combine novel treatments is considered.

The optimal strategy to treat patients with more established CKD also requires clarification. Depending on the country, a high proportion of patients may be diagnosed at later stages of the disease, where significant irreversible nephron loss may have already occurred.^1^^,^^6^ Among patients with IgAN, 35% to 75% present with eGFR <60 ml/min per 1.73 m^2^ at diagnosis, with a high proportion of patients (29%) presenting with <29 ml/min per 1.73 m^2^ at diagnosis in the United States (Sim JJ, Chen Q, Chang JM, et al. ESKD and CKD progression among a diverse immunoglobulin A nephropathy [IgAN] population [abstract]. American Society of Nephrology Kidney Week. November 1–5, 2023; Philadelphia, PA. Abstract TH-PO615).^1^^,^^6^^,^^106^^,^^107^ However, there are limited data in patients with eGFR <30 ml/min per 1.73 m^2^, because these patients are seldom included in randomized clinical trials because of their advanced kidney function decline.^11^ Further investigation is needed to determine whether existing therapies have an impact on clinical course at advanced stages of the disease, particularly as pathologic features, such as scarring and fibrosis, may be difficult to reverse.^8^^,^^108^

### Unique IgAN populations with the greatest unmet needs

IgAN also impacts pediatric populations, with annual incidences of 0.20/100,000 and 9.9/100,000 reported across 6 European countries and Japan, respectively.^109^^,^^110^ Currently, every large randomized clinical trial investigating emerging therapies has included only adult patients aged ≥18 years.^11^^,^^21^^,^^23^^,^^25^ Currently, there is only 1 phase 2 trial (NCT05003986, Study of Sparsentan Treatment in Pediatrics With Proteinuric Glomerular Diseases [EPPIK]) that is recruiting pediatric patients with IgAN to evaluate the safety, efficacy, and tolerability of sparsentan over 108 weeks.^111^ Further work in this population of patients is required to understand if emerging therapies are of benefit.

The recurrence of IgAN in patients post-transplant represents another unmet need. It is recognized that despite excellent earlier graft function, clinical recurrence of IgAN post-transplant impacts graft survival and is an important cause of graft loss.^112^ These patients have been largely excluded from recent clinical trials. There are unique questions to be addressed in this population, such as the safety of adding another arm of immunosuppression and the risks or benefits associated with additional manipulation of glomerular hemodynamics in patients with a single denervated kidney. Finally, there are no approved targeted therapies for patients with IgA vasculitis or rapidly progressive variants of IgAN. These studies are difficult to design and execute because of the rarity of the condition, but this also represents an important unanswered question and unmet need.

### Access to future medicines

Accessibility of therapies is always a concern when new agents are approved, especially in countries where the burden of IgAN is highest. Cost-effectiveness analysis data demonstrating the benefits of treatment, such as reduction of overall health care costs and ability of patients to continue to engage in daily activities and have a good quality of life, are required. For example, a cost-effectiveness model in the United States, comparing Nefecon plus supportive care versus supportive care alone, estimated that Nefecon use would remain cost-effective for up to 4 9-month courses of treatment and provide patients with a better state of health.^113^ Further cost-effectiveness research across different approved interventions that become available in the future would be beneficial to improve access to medicines and help guide treatment selection. However, such cost-effectiveness research alone is insufficient given the global burden of IgAN-induced kidney failure. Efforts to provide equitable access to highly effective therapies, particularly when layering multitarget treatment approaches and in resource-limited countries and/or publicly funded health care systems, should remain a priority.

## Conclusions

Given the swift pace of change in the IgAN treatment landscape, we expect current and future IgAN treatment guidelines to evolve just as swiftly to reflect the availability of novel agents that treat IgAN (Figure 1b). As guidelines evolve, it is important to remember the value of individualized patient care by considering medical history and personal circumstances and preferences, and involving patients in the shared decision-making process.

The identification of patients most likely to benefit from specific treatments requires further investigation, possibly incorporating a patient’s demographic and clinical characteristics, histology, biomarkers, and other validated prognostic markers. Further understanding is needed on several aspects of IgAN management, such as the choice and the optimal duration of different treatments, cyclical or maintenance treatment approaches, safety of combination therapy, and treatment efficacy and safety in certain patient populations, such as pediatric patients. Although substantial costs are associated with drug development and the aforementioned additional research needed in IgAN, it is critical that efforts continue in improving the care of patients and ensuring widespread access to new medicines.

## Disclosure

This article is published as part of a supplement sponsored by Calliditas Therapeutics, an Asahi Kasei company.

SCWT reports consulting fees from Boehringer Ingelheim, Novartis, and Travere Therapeutics; honoraria from AstraZeneca, Alexion, Bayer, Boehringer Ingelheim, CSL Vifor, Everest Medicines, GSK, Novartis, and Vera Therapeutics; and having been an Executive Committee Member of KDIGO from 2020 to 2023 and a coauthor of the 2025 KDIGO Clinical Practice Guidelines for the Management of Immunoglobulin A Nephropathy and Immunoglobulin A Vasculitis. HNR has received grant support from the Canadian Institutes of Health Research and the Kidney Foundation of Canada (from John and Leslie Pearson); has received research expenses (not personal fees) for clinical trials from Alexion, Calliditas Therapeutics, Novartis, and Omeros; reports consulting fees, honoraria, or travel support from Alexion, Biogen, Calliditas Therapeutics, Chinook, Novartis, Omeros, Otsuka, Pfizer, Travere Therapeutics, and Vera Therapeutics; has served on advisory boards and steering committees for Alexion, Chinook, Novartis, Omeros, Otsuka, Pfizer, and Travere Therapeutics; has been an investigator for Alexion, Alnylam Pharmaceuticals, Calliditas Therapeutics, ChemoCentryx, Chinook, Novartis, Omeros, and Pfizer; and is director of the Glomerulonephritis Fellowship, supported by the Louise Fast Foundation and Otsuka Canada. She served as a coauthor of the 2025 KDIGO Clinical Practice Guidelines for the Management of Immunoglobulin A Nephropathy and Immunoglobulin A Vasculitis.
